# Electrochemical and Photoelectrochemical Properties of Nickel Oxide (NiO) With Nanostructured Morphology for Photoconversion Applications

**DOI:** 10.3389/fchem.2018.00601

**Published:** 2018-12-12

**Authors:** Matteo Bonomo, Danilo Dini, Franco Decker

**Affiliations:** Department of Chemistry, University of Rome La Sapienza, Rome, Italy

**Keywords:** solar energy conversion, photoelectrochemistry, photoelectrochemical cells, semiconductor nanostructures, metal oxide nanostructures, nickel oxide nanoparticle

## Abstract

The cost-effective production of chemicals in electrolytic cells and the conversion of the radiation energy into electrical energy in photoelectrochemical cells (PECs) require the use of electrodes with large surface area, which possess either electrocatalytic or photoelectrocatalytic properties. In this context nanostructured semiconductors are electrodic materials of great relevance because of the possibility of varying their photoelectrocatalytic properties in a controlled fashion via doping, dye-sensitization or modification of the conditions of deposition. Among semiconductors for electrolysers and PECs the class of the transition metal oxides (TMOs) with a particular focus on NiO interests for the chemical-physical inertness in ambient conditions and the intrinsic electroactivity in the solid state. The latter aspect implies the existence of capacitive properties in TMO and NiO electrodes which thus act as charge storage systems. After a comparative analysis of the (photo)electrochemical properties of nanostructured TMO electrodes in the configuration of thin film the use of NiO and analogs for the specific applications of water photoelectrolysis and, secondly, photoelectrochemical conversion of carbon dioxide will be discussed.

## Introduction

Semiconductors (SCs) have been considered as electrode materials since the late fifties when a photopotential (*V*_photo_) could be generated by an electrochemical cell employing either *p*- or *n*-type Ge electrodes (Brattain and Garrett, 1955). In more recent years, SC electrodes in the nanostructured version (Hagfeld and Grätzel, 1995) were considered because of the modification of the scheme of energy levels at the SC/electrolyte interface in passing from the bulk/compact version to the nanostructured one. In fact, nanostructured SCs present isolated energy levels in the intragap region, which are associated to localized/confined states (Brus, 1986) and possess a relatively larger density of surface states with respect to bulk states when compared to compact SCs (Hagfeld and Grätzel, 1995). Moreover, the open morphology renders the nanostructured SCs particularly active in the realization of those interfacial processes which require a large contact area due to the presence of large voids on the surface. When the nanostructured SC possesses a wide bandgap of a given width *h*ν_1_, (Gerischer et al., 1968) this is more prone to an efficacious action of sensitization at lower optical energies *h*ν_2_ (with *h*ν_2_<*h*ν_1_), upon anchoring of an opportune sensitizer due to the very large surface concentration of active sites on which sensitization takes place (Gibson et al., 2013; Bonomo et al., 2016a). The interest about the nanostructured SC sensitization has boosted tremendously after the advent of the dye-sensitized solar cell (DSC), (O'Regan and Gratzel, 1991) i.e., a photoelectrochemical cell (PEC) the working principle of which consists in the dye-mediated *et* between the wide bandgap SC in the sensitized state and a redox couple with equilibrium energy level *E*_0, r_ positioned within the SC bandgap.

The considerable success of this approach (Ito et al., 2008; Yella et al., 2011; Mathew et al., 2014; Kakiage et al., 2015b) is witnessed by the quite high overall efficiencies of light-to-current conversion (η_DSC_), for which the present record is *ca*. 14% (Kakiage et al., 2015a). DSCs present high photoelectroactivity per unit area as imparted by the mesoporous morphology of the surface of the sensitized SC electrode. More recently, nanosized SCs electrodes have been considered for the development of useful and economically competitive photoelectrochemical reactors (or photoelectrolysers; Brennaman et al., 2016) for the conversion of the solar radiation into chemicals of high energetic value (e.g., production of non-fossil fuels like molecular hydrogen from solar water splitting; Li et al., 2012, 2015; Mayer et al., 2013; Dotan et al., 2014; Antila et al., 2016; Hoogeveen et al., 2016). Nanostructured SC electrodes have been also considered for the realization of photoactivated electrochemical processes with consumption of reactants having strong environmental impact (e.g., the light-activated reduction of carbon dioxide into fuels or synthons). For the great relevance of the three main applications of electrical power production from the conversion of sunlight, solar generation of H_2_ fuel from water splitting, and solar-driven photoreduction of CO_2_ with photoelectrochemical devices utilizing nanostructured SC electrodes as photoelectroactive components (either in the bare or in the sensitized state), it appears important to analyse and review the recent developments in the materials science behind the design, production and characterization of semiconducting electrodes with characteristic size of 10^−9^ m. In particular, the present contribution will focus on the analysis of the electrochemical and photoelectrochemical properties of nanostructured SCs made of transition metal oxides (TMOs) with particular attention to NiO (He et al., 1999; Cerc Korošec et al., 2003; Nakasa et al., 2005; Hongjun et al., 2007; Awais et al., 2010, 2013b, 2014; Deng et al., 2012; Powar et al., 2012; Pan et al., 2013; Qu et al., 2013; D'Amario et al., 2014; Flynn et al., 2014; Huang et al., 2014; Wang et al., 2014; Nail et al., 2015; Naponiello et al., 2015; Zannotti et al., 2015; Battiato et al., 2016; Li X. et al., 2016; Wei et al., 2016; Wood et al., 2016; Di Girolamo et al., 2018). A typical feature of semiconducting TMOs in the configuration of nanostructured thin films (thickness *l* < 50 μm), is the possibility of varying electrochemically/photoelectrochemically the redox states of their constituting units, i.e., metal centers and/or oxygen anions (Hagfeldt et al., 1994; Ma et al., 2014; Marrani et al., 2014). The electronic properties of nanostructured SC-TMOs like bandgap width, optical absorption, charge carrier concentration/mobility and flatband potential can be then modulated opportunely through electrochemically/photoelectrochemically driven processes. In the following the aspects of the electrochemical and photoelectrochemical properties of nanostructured SCs based on NiO and their employment in photoelectrochemical devices of practical interest will be considered.

## Electrochemical Properties of Nanostructured NiO

Nanostructured SC electrodes made of TMOs with one (Rettie et al., 2016) or more metal atoms (Rowley et al., 2014; Sullivan et al., 2015; Jiang et al., 2016a) can be prepared and deposited in many ways utilizing either a chemical approach (Boschloo and Hagfeldt, 2001; Li et al., 2010; Venditti et al., 2014) or physical methods for the attainment of electrodes in the configuration of thin film (Passerini et al., 1993; Twomey et al., 2008; Awais et al., 2010, 2013a; Gibson et al., 2013; McDonnell et al., 2013; Bonomo et al., 2016a). These include sol-gel procedures(Boschloo and Hagfeldt, 2001; Li et al., 2010), template chemistry, screen-printing (Twomey et al., 2008; Gibson et al., 2013; Bonomo et al., 2016c), plasma assisted microwave sintering (McCann et al., 2011; Awais et al., 2013a; McDonnell et al., 2013), micropowder microblast, (Awais et al., 2013a; McDonnell et al., 2013) magnetron sputtering (Passerini et al., 1993; Awais et al., 2010; McCann et al., 2011; McDonnell et al., 2012) and electrodeposition (Venditti et al., 2014) among others. The most common examples of nanostructured photoelectroactive SCs of *n*-type are TiO_2_ in the anatase form Wu et al. (2008), hexagonal ZnO, (Rensmo et al., 1997; Keis et al., 2001, 2002; Boucharef et al., 2010; Dupuy et al., 2010; Tian et al., 2011; Li M. et al., 2016) Fe_2_O_3_ hematite, (Kay et al., 2006; Congiu et al., 2017) WO_3_, (Masetti et al., 1995; Dini et al., 1996) VO_x_ (Wang et al., 2006; Sai Gautam et al., 2016) and Nb_2_O_5_ (Fiz et al., 2016). Semiconducting TMOs of *p*-type (Bonomo and Dini, 2016) include cubic NiO, (He et al., 1999; Cerc Korošec et al., 2003; Nakasa et al., 2005; Hongjun et al., 2007; Powar et al., 2012; Awais et al., 2013b, 2014; Pan et al., 2013; Qu et al., 2013; Flynn et al., 2014; Nail et al., 2015; Naponiello et al., 2015; Battiato et al., 2016; Li X. et al., 2016; Wood et al., 2016) Li-doped NiO,(Wang et al., 2014; Wei et al., 2016) mixed Ni and Mg oxides, (Deng et al., 2012; D'Amario et al., 2014; Huang et al., 2014; Zannotti et al., 2015) Cu_2_O (Jiang et al., 2016b), CuMO_2_ (Yu et al., 2014) and derivatives of LaO_x_ (Renaud et al., 2015). The methods of preparations of nanostructured SCs can affect considerably the electrochemical properties of the differently deposited materials despite the same nominal chemical composition. At this regard, the most striking example is given by nanostructured NiO, i.e., the most widely employed metal oxide of *p*-type for advanced energy conversion/storage applications, for which the comparative characterization of the electrochemical and photoelectrochemical properties has shown a clear evidence of the influence of the preparation/deposition method on the charge transport/transfer properties of the resulting thin film NiO electrodes (Cerc Korošec et al., 2003; Nakasa et al., 2005; Powar et al., 2012; Awais et al., 2013a,b, 2014, 2015a,b; Gibson et al., 2013; Qu et al., 2013; Naponiello et al., 2015; Wood et al., 2016). Some semiconducting TMOs are electroactive in the solid state. This is equivalent to say that SC-TMOs can alter the oxidation state(s) of the constituting metal centers and oxygen atoms by means of redox processes that are electrochemically driven (Hagfeldt et al., 1994; Boschloo and Hagfeldt, 2001; Awais et al., 2013b; Marrani et al., 2014). Electroactivity thus represents an intrinsic property of SC-TMOs, which implies the imparting of charge storage properties in these systems (Estrada et al., 1988; Passerini et al., 1990; Decker et al., 1992; Passerini and Scrosati, 1992; Kitao et al., 1994; Talledo et al., 1994; Gökdemir et al., 2014; Lykissa et al., 2014; Wen et al., 2014). The TMOs electroactivity is granted in those oxides in which the metal atom can present more than one stable redox state (Passerini and Scrosati, 1994; Bellakhal and Draou, 1997; Grugeon et al., 2001; Dong et al., 2003; Sudant et al., 2004; Wang et al., 2005; Mjejri et al., 2014; Vernardou et al., 2014) but it does not arise from nanostructured morphology (Sun and Tolbert, 2004; Centi and Perathoner, 2009; Poppe et al., 2014). Mesoporosity, in turn, would affect mainly the surface concentration of defects, (Liu D. et al., 2011; Liu Y. et al., 2011; Uchaker et al., 2015) the kinetics of metal oxide electrochemistry, (Weng et al., 2013; Zhang et al., 2013) the extent of exchangeable charge during electrochemical processes, (Spahr et al., 1998, 1999; Dobley et al., 2001; Nordlinder et al., 2006) and the rate of the eventual chemical dissolution processes accompanying these redox reactions (Marrani et al., 2014). Among TMOs of *p*-type, nickel oxide(s)(Dini et al., 2015a) and vanadium oxide(s)(Coustier, 1999; Li et al., 2006; Choi et al., 2009) the systems with the more complex electrochemical behavior due to the existence of stable binary oxides with the metal centers possessing more than two oxidation states. An important feature of the electrochemical switching of TMOs redox states is the reversibility (Passerini et al., 1990). Moreover, the electrochemical switching of the redox states in TMOS can lead to the formation of non-stoichiometric oxides due to the co-existence of metal centers having different formal oxidation numbers. A particularly interesting electrodic material that is amenable to act as a photoelectrocatalytic agent in photoactivated processes of reduction is NiO in the nanostructured form and in the sensitized state (Dini et al., 2015a; *vide infra*). This is due to the richness of the electrochemical and photoelectrochemical behavior of nanostructured NiO as proved by its direct participation in reversible processes of oxidation and reduction of various nature.

### Oxidation Processes

**Figure 1:**
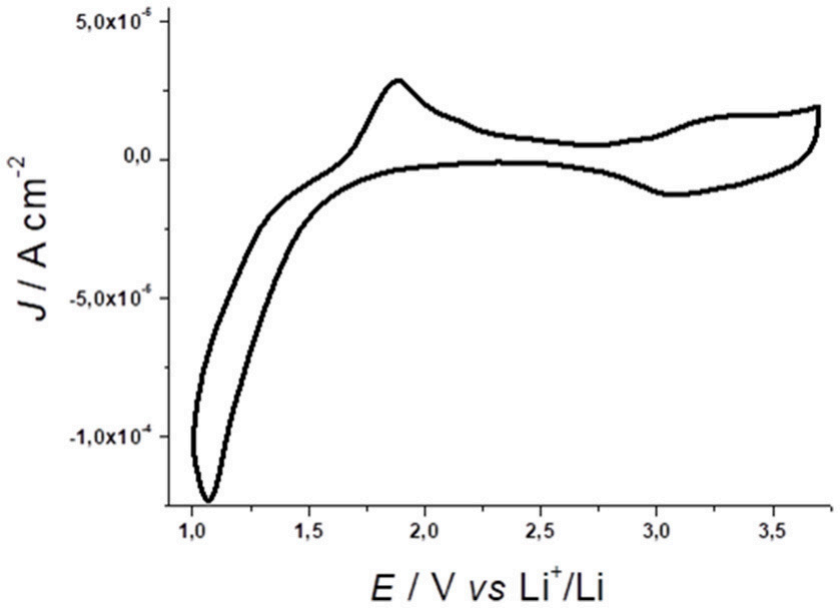
Cyclic voltammetry of NiO prepared via conventional sintering of sprayed NiO nanoparticles (average diameter, ø: 50 nm). Electrolyte composition: 0.7M LiClO_4_ in anhydrous propylene carbonate; counter electrode: Li; reference electrode: Li^+^/Li; scan rate: 15mV s^−1^. NiO thickness: 0.3 μm. Adapted from Awais et al. (2013b).

Nanostructured NiO thin films (*l* < 5 μm) in non-aqueous electrolytes and in anhydrous/anaerobic atmosphere generates the voltammetric profile of Figure 1 (Awais et al., 2013b). Nickel Oxide (NiO) is a green crystalline solid material with ferromagnetic properties (Neel temperature is 523 K) NiO have unique electrical, magnetic and optical properties that make it the main subject of a considerable number of scientific papers. NiO is a wide band gap [3.6–4.0 eV(Wang et al., 2015)] p-type semiconductor and it experimented an extreme photochemical, electrochemical and physical stability. Both optical and electronic properties of NiO depends on its degree of defectivity. As a matter of fact, NiO should be better described by the formula NiO_x_ in which x accounts for the presence of Ni(III) site in the matrix of the nickel oxide. The latter became an interesting material of research due to its low cost and excellent ion storage property. For example, NiO nanostructures are p-type semiconductors with peculiar magnetic and electric behavior depending on the particle size. A comprehensive analyses of the different nanostructured morphologies, in which NiO could be obtained, falls outside the purpose of the present review work. Furthermore, a recent review paper by Bonomo properly faced this topic (Bonomo, 2018).

The oxidation processes verified at *E* > 2.5 V vs. Li^+^/Li (Figure 1) are ascribed to two redox reactions consisting formally in the nickel based oxidations (Awais et al., 2013b)

(1) $$\begin{array}{r} \text{NiO}+\text{m}X^{-}\to {\text{Ni}\left(\text{II}\right)}_{1-\text{m}}{\text{ONi}\left(\text{III}\right)}_{\text{m}}{\left(\text{X}\right)}_{\text{m}}+{\text{m e}}^{-} \end{array}$$

$$\begin{array}{r} {\text{Ni}\left(\text{II}\right)}_{1-\text{m}}{\text{ONi}\left(\text{III}\right)}_{\text{m}}{\left(\text{X}\right)}_{\text{m}}+\text{m}{\text{X}}^{-}\to {\text{Ni}\left(\text{II}\right)}_{1-\text{m}} \end{array}$$

(2) $$\begin{array}{r} \;{\text{ONi}\left(\text{IV}\right)}_{\text{m}}{\left(\text{X}\right)}_{2\text{m}}+{\text{m e}}^{-} \end{array}$$

in which X^−^ is a singly charged anion that acts as charge-compensating species. Equations 1 and 2 refer, respectively, to the nickel based oxidations Ni(II) → Ni(III) and Ni(III) → Ni(IV), with the first occurring at lower potential values (Marrani et al., 2014). It is worth to mention that both the above-reported equation shall remain valid both in an aqueous and organic environment. The main difference consists in the nature of the anions compensating the positive charges produced in NiO matrix: in the former case it is a hydroxyl anion (i.e., OH-), in the latter it is an anion present in the electrolyte (e.g., ClO_4_^−^ in a LiClO_4_ ACN solution).

As a matter of fact, Ni(III) centers can be already present in the pristine nanostructured oxide (Marrani et al., 2014; Bonomo et al., 2016b) since the open circuit potential of the cells with NiO working electrodes generally surpasses the value of the potential onset for NiO oxidation (*E*_onset_ = 2.5 V vs. Li^+^/Li, Figure 1; Awais et al., 2010; Naponiello et al., 2015; Sheehan et al., 2015; Bonomo et al., 2016a). The oxidation processes 1 and 2 manifest themselves through two broad peaks with generally different amplitude. At the microscopic level these oxidation processes are accompanied by a variation of the NiO electrode mass for the transfer of the charge-compensating anions X^−^ (Equations 1 and 2) from the electrolyte to the electrode. This kind of process is generally accompanied by the production of mechanical stress in the electrode (Dini and Decker, 1998). Upon continuous electrochemical cycling the electrode tends to minimize such a mechanical stress by modifying and re-arranging its surface structure and morphology (Awais et al., 2015b; Bonomo et al., 2018c). This structural evolution of the NiO electrode is commonly accompanied by the progressive increase of the amount of charge that NiO electrode is capable to exchange, host and store during oxidation (Figure ESI1; Awais et al., 2013b). Upon stabilization of the voltammogram, the system undergoes a process of oxidation which is surface confined as evinced by the linear the relationship between current peak and scan rate (Figure ESI2; Sheehan et al., 2015). NiO electrodes can sustain up to several hundreds of electrochemical cycles in non-aqueous electrolyte thus proving an appreciable chemical and physical stability (Novelli et al., 2015). Recently Bonomo et al. reports on the ameliorated electrochemical stability in aqueous environment (Bonomo et al., 2018b). In that paper, the authors evidenced how the insufficient stability of NiO is mainly due to the occurrence of degradation processes catalyzed by the presence of chloride and perchlorate anions. The presence of these two anions could be easily avoided in photoelectrochemical devices by the control of the nature of the supporting electrolyte. Furthermore, applied potential lower than 0.8 V vs. Ag/AgCl does not cause any degradation of the electrode.

The nanostructured feature of the electroactive NiO electrode manifests itself electrochemically through the observation of the direct proportionality between exchanged current and NiO electrode thickness during the oxidation processes of Equations 1 and 2 (Figure ESI3; Novelli et al., 2015). The correlation between NiO film thickness and amount of exchanged charge retains its validity when one refers to nanostructured NiO samples that have been prepared with the same methodology an d are electrochemically cycled under analogous experimental conditions (Awais et al., 2011; Gibson et al., 2013). In fact, it has been verified that NiO electrodes with the same nominal thickness can vary considerably the amount of exchanged charge during NiO oxidation if these films have been deposited with different methods or prepared from different precursors (Gibson et al., 2013; Naponiello, 2015; Bonomo et al., 2016a,b; Wood et al., 2016). This is because the method of deposition/preparation can severely affect the surface area, the surface chemical composition, the electrical connectivity between the nanoconstituents of NiO film, and the level of adhesion between the NiO deposit and the conductive transparent substrate (Awais et al., 2013a, 2014; Gibson et al., 2013). Another factor that can modify the shape of the voltammograms of NiO during electrochemical oxidation is the composition of the electrolyte (Figure ESI4; Novelli et al., 2015), which imposes the mechanisms of charge compensation/mass transfer simultaneous to NiO oxidation: the variability of ion sizes, ionic adsorption, and eventual ion coupling on the surface of the nanostructured electrode are all phenomena-characteristics that might vary considerably the kinetics and thermodynamics of charge-mass transfer processes at the NiO electrode/electrolyte interface (Gregg, 2004).

NiO electrochemical oxidation is not sensitive to light irradiation as demonstrated by the invariability of the voltammogram in passing from the dark condition to the illuminated one with a white light lamp as source of luminous radiation (Novelli et al., 2015). Therefore, the oxidation of nanostructured NiO is a redox process which does not get activated photoelectrochemically despite the fact that NiO has a broad featureless absorption and possesses electrochromic activity in the visible range (Boschloo and Hagfeldt, 2001; Granqvist, 2012). The electrochemical oxidation of NiO can be sensitive to the luminous radiation if the oxide is dye-sensitized (Figure ESI5; Gibson et al., 2013; Awais et al., 2014, 2015a,b). The resulting effect is the increase of the photoconductivity of the modified electrode obtained by the combination dye-sensitizer/NiO with the redox processes that are based exclusively on the electroactivity of NiO substrate. In absence of illumination dye-sensitization of nanostructured NiO generally provokes an effect of surface passivation as far as the process of NiO oxidation is concerned with the observation of a diminution of the electrical current associated to the NiO-based redox reaction (Gibson et al., 2013; Awais et al., 2014). The extent of current diminution depends on the nature of the colorant and on the conditions of sensitization (Sheehan et al., 2015). With the exception of squaraine-sensitized NiO (Naponiello et al., 2015; Sheehan et al., 2015; Bonomo et al., 2016a), the voltammogram of which displays also redox peaks characteristic of the anchored squaraine (Figure ESI6), it is found generally that the most common colorants of NiO do not display electroactivity within the potential range of NiO oxidation (Bonomo et al., 2018a). Alike NiO in the bare state (Figure ESI1), the electrochemical oxidation of NiO sensitized with erythrosine b, N719, black dye, or P1 (Gibson et al., 2013; Awais et al., 2014) involves an initial process of electrode activation (Figure ESI7, left frame), and shows a linear variation of the current peaks with the scan rate (Figure ESI7, right frame). A useful parameter for the analysis of the interfacial properties of dye-sensitized NiO electrodes is the open circuit potential (OCP) of the cell with NiO working electrodes. The comparison of the OCP values nanostructured NiO in the pristine and sensitized states shows that the presence of the dye tends generally to lower the OCP with respect to bare NiO electrode (Table 1; Morandeira et al., 2005, 2008; Hongjun et al., 2007; Awais et al., 2013b, 2015a,b; Gibson et al., 2013; Naponiello et al., 2015; Sheehan et al., 2015; Bonomo et al., 2016c). The change of OCP provoked by the dye-sensitizer is indicative of the variations of the electrical potential at the NiO electrode/electrolyte interface and the lowering of OCP in passing from the bare to sensitized state is a straightforward consequence of the diminution of positive charge (or increase of negative charge) on the surface of the sensitized electrode with respect to its pristine state. Therefore, sensitization is accompanied by a concomitant process of charge transfer between the dye and the electrode. Sensitization certainly alters the surface density of the charge and, at the microscopic level, the observed diminution of OCP can correspond to the neutralization of the positively charged surface sites of pristine NiO as determined with XPS (Bonomo et al., 2017). Another plausible mechanism of OCP diminution following dye-sensitization is the coverage of the positive charges exposed on NiO surface by the chemisorbed dye-sensitizer which would act thus like a depolarizer or electrical insulator. Sensitization also modifies the tendency of the ions of a given electrolyte to adsorb on the electrode surface. Consequently, the decrease of OCP can originate also from the fact that the sensitizer favors somehow the adsorption of negative ions from the electrolyte on the surface of sensitized NiO with respect to the situation generated by the bare electrode of NiO (Bonomo et al., 2018a). Finally, another mechanism at the basis of OCP diminution could be the loss of a positive charge by the sensitizer once it gets adsorbed onto NiO surface (example of sensitizer deprotonation upon anchoring).

**Table 1:** Values of open circuit potential of the cells with screen-printed NiO in the bare or sensitized state as working electrode.

**NiO sample**	**Dye-sensitizer**	**Open circuit potential/V vs. Ag/AgCl**
**1**	-	0.113
**1**	Erythrosine b^*^	−0.144
**1**	Erythrosine b^**^	0.028
**1**	Cumarin 343	0.280
**1**	Cumarin 153	0.150
**1**	Fast Green	−0.055
**1**	P1	0.140
**2**	–	0.360
**2**	VG1	0.150
**2**	VG10	−0.210
**2**	VG11	−0.187
**2**	DS35	0.005
**2**	DS44	−0.160
**2**	DS46	−0.243
**3**	–	0.340
**3**	VG11	0.142
**3**	DS46	0.110
**3**	Fast Green	0.040

*Electrolyte: 0.2 M LiClO_4_ in 3-methoxy-propionitrile. Sample **1** refers to NiO deposits screen printed from slurries with 1 mL of HCl. Samples **2** and **3** refer to NiO deposits screen printed form slurries with 1 mL and 5 mL of CH_3_CO_2_H, respectively*.

* NiO sample immersed in the sensitizing solution for 2 h

** *NiO sample immersed in the sensitizing solution for 16 h*.

It has been found that sensitized NiO under illumination (Figure ESI8) follows a kinetics of oxidation which is analogous to what has been observed for the same process occurring at the pristine oxide (Figure ESI2, left plot) and at its dye-sensitized version in dark conditions (Figure ESI7, right plot; Awais et al., 2013b).

The linear dependence of the current peaks on scan rate (Figure ESI8) is the further confirmation that the processes of NiO oxidation in Equations 1 and 2 are kinetically limited by the step of charge transfer at the electrode/electrolyte interface (Gregg, 2004) no matter whether NiO is sensitized or not (Boschloo and Hagfeldt, 2001; Novelli et al., 2015). When an aqueous electrolyte is used (Boschloo and Hagfeldt, 2001; Gibson et al., 2013) the process of oxidation of nanostructured NiO presents again the linearity of the current intensity peaks with the scan rate (Boschloo and Hagfeldt, 2001; Marrani et al., 2014), and the direct proportionality of the amount of exchanged charge with film thickness (Gibson et al., 2013). Also in aqueous electrolytes the shape of the voltammogram depends on the methods of preparation and deposition of nanostructured NiO (Gibson et al., 2013; Wood et al., 2016).

The peculiarity of the electrochemistry of nanostructured NiO electrodes in water based electrolytes is the verification of the oxide dissolution upon repetitive cycling (Marrani et al., 2014). The reactions that lead to the chemical dissolution of NiO during its electrochemical oxidation are:

(3) $$\begin{array}{r} {\text{N}{\text{i}}^{\text{II}}\text{O}\left(\text{OH}\right)}_{n}{\left({\text{H}}_{2}\text{O}\right)}_{p}\to {\text{N}{\text{i}}^{\text{II}}\text{N}{\text{i}}^{\text{III}}\text{O}\left(\text{OH}\right)}_{n+1}{\left({\text{H}}_{2}\text{O}\right)}_{p-1}+e^{-}+{\text{H}}^{+} \end{array}$$

for the oxidative process associated to the current peak I (see indexing of the oxidation peaks in Figure 4a of Boschloo and Hagfeldt (2001), and

(4) $${\text{Ni}}^{\text{II}}{\text{Ni}}^{\text{III}}\text{O}{\left(\text{OH}\right)}_{n+1}{\left({\text{H}}_{2}\text{O}\right)}_{p-1}\to {\text{Ni}}^{\text{II}}{\text{Ni}}^{\text{IV}}\text{O}{\left(\text{OH}\right)}_{n+2}{\left({\text{H}}_{2}\text{O}\right)}_{p-2}+e^{-}+{\text{H}}^{+}$$

for the oxidation process associated to peak II (see indexing of the oxidation peaks in Figure 4a of Boschloo and Hagfeldt (2001). In Equations 3 and 4 the metal oxide is formulated as a hydrated system upon contact of the oxide with the aqueous electrolyte. As previously outlined, the Ni centers that undergo oxidation according to Equations 3 and 4 are localized on the surface of the oxide. Chemical dissolution of the electrode would be partially due to the progressive transformation of the insoluble oxide(s) into water-soluble soluble hydroxide(s).

**Figure 2:**
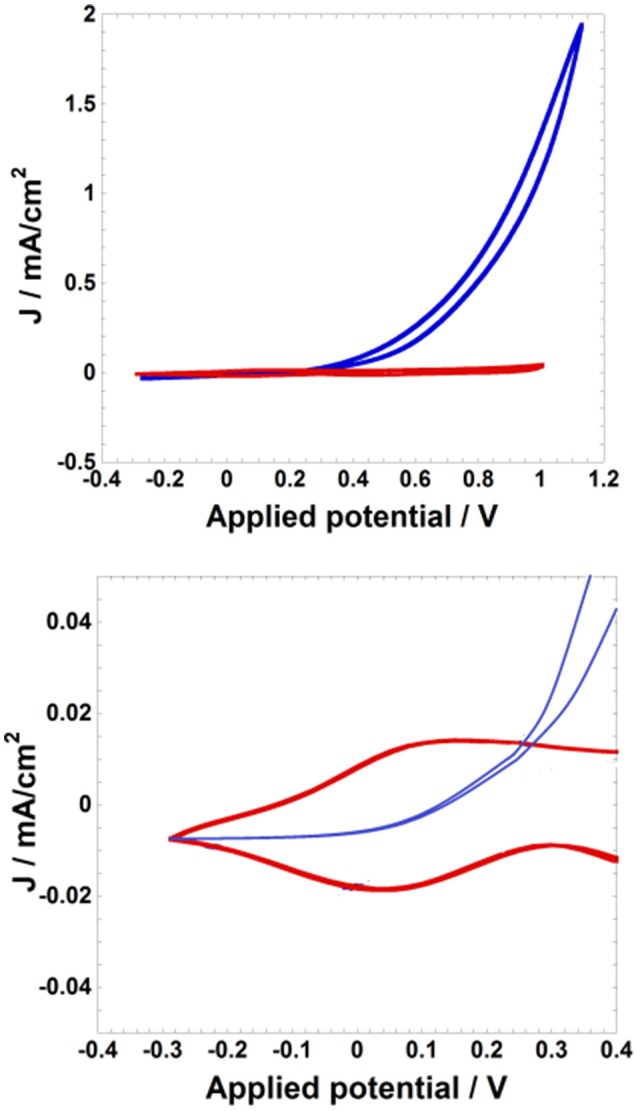
Cyclic voltammetries of a NiO film (*l*: 4 μm) at the scan rate of 10mV s^−1^ in two different electrolytes (red curve: 0.2M LiClO_4_ in acetonitrile; blue curve: 0.2M LiI and 0.02 M I_2_ in acetonitrile). Potential values are referred to the redox couple Ag/AgCl. Top: voltammograms in the full scale of current; bottom: zoom of the two voltammograms in correspondence of the onset of NiO (red profile) and I^−^ (blue profile) oxidations. Reproduced with permission from Bonomo et al. (2016b).

Oxidation of nanostructured NiO can impart electroctalytic properties to NiO toward the oxidation of redox active species (vide supra; Sheehan et al., 2015; Bonomo et al., 2016b). This is the case of the conversion of I^−^ to I${}_{3}^{-}$ (the typical anodic process of a DSC or one of its recombination reactions; Boschloo and Hagfeldt, 2009) occurring at electrochemically oxidized NiO (Bonomo et al., 2016b). The latter species assumes the electrical potential and increases correspondingly its electrical conductivity to accomplish the reaction of oxidation of I^−^ (Figure 2).

The current exchanged by oxidized NiO for the transformation of iodide to tri-iodide is of an order of magnitude larger than the current of NiO oxidation (Figure 2). Moreover, it results that the potential threshold for the onset of iodide oxidation is slightly larger than the potential of NiO oxidation itself (Figure 2). This sequence of events is indicative of the switching of the NiO electrode into an active state toward I^−^ oxidation through the electrochemical injection of holes in NiO. For the disambiguation of the possible intervention of the FTO susbtrate as actual electrodic material of I^−^ oxidation in case FTO is not covered uniformly by the layer of nanoporous NiO, some authors have analyzed comparatively the redox process of I^−^ oxidation on bare FTO electrodes and on NiO-covered FTO substrates (Sheehan et al., 2015; Bonomo et al., 2016b) when NiO had nanoporous features. It was verified that nanostructured NiO films in the oxidized state displayed generally an electrocatalytic effect on I^−^ oxidation with respect to bare FTO substrate (Figure ESI10). This is because the onset of potential of iodide oxidation was systematically lower on nanoporous NiO electrodes deposited onto FTO substrates with respect to the uncovered FTO substrate (Figure ESI10). In turn, the method of preparation of the thin film of nanostructured NiO had an influence on the resistance of *et* associated to the oxidation 3I^−^ → I${}_{3}^{-}$ + 2 *e*^−^ at the NiO electrode/electrolyte interface. This was evinced by the different slopes of the voltammetric curves generated with the diversely deposited NiO electrodes (comparison of the voltammograms generated by NiO in the two different plots of Figure ESI9). The film of nanoporous NiO prepared via plasma-assisted rapid discharge sintering (Awais et al., 2011) resulted electrochemically more efficient since it presented a faster kinetic of *et* (Figure ESI9, left plot) with respect to screen-printed NiO (Naponiello et al., 2015; Bonomo et al., 2016c) as far as the oxidation process 3I^−^ → I${}_{3}^{-}$ + 2 *e*^−^ is concerned (Figure ESI9, right plot). This is ascribed to the better electrical connectivity between sintered NiO nanoparticles and between the NiO film and the FTO substrate in the samples obtained via plasma assisted sintering (Awais et al., 2011, 2014; Gibson et al., 2013; Sheehan et al., 2015) with respect to the screen-printed version of nanoporous NiO electrodes (Bonomo et al., 2016b).

### Reduction Processes

NiO electrodes can undergo solid-state reduction in a reversible manner when are immersed in an electrolytes (Awais et al., 2010, 2013a,b). Under these circumstances the electrochemical reaction of cation intercalation occurs according to:(Passerini et al., 1990; Decker et al., 1992; Owens et al., 1999; Wang and Cao, 2006).

(5) $$\begin{array}{l} \text{NiO}+{\text{xe}}^{-}+\text{x}{\text{M}}^{+}\to {\text{M}}_{\text{x}}\text{NiO} \end{array}$$

In Equation 5 M^+^ represents a singly charged cation of small radius (usually Li^+^ or H^+^). The process of Equation 5 corresponds to the electrochemical *n*-doping of NiO. It has been verified that electrochemical reduction of NiO affects its electrical conductivity, optical absorption, ionic conduction and magnetic properties (Passerini and Scrosati, 1994). Alike solid-state oxidation (Figure ESI1), the solid-state reduction of nanostructured NiO goes through a process of electrode activation consisting in the progressive increase of the amount of exchanged charge upon repetition of electrochemical cycles (Figure ESI10). At a microscopic level such an electrochemical activation of the reaction of NiO reduction corresponds to the aperture/enlargement of channels of cation intercalation within the oxide structure (Passerini et al., 1990; Decker et al., 1992; Passerini and Scrosati, 1994). Different to oxidation, the electrochemical reduction of NiO follows a kinetics which is controlled by diffusion (Bard and Faulkner, 2001). This was verified through the linear dependence of the height of the reduction current peak on the square root of the scan rate after stabilization of the voltammogram of NiO (Awais et al., 2013a). Impedance spectra also indicated the presence of relevant diffusive phenomena for NiO in passing from the neutral pristine state to the fully reduced state through the observation of more pronounced capacitive features in Li_*x*_NiO at the lowest frequencies of potential stimulus (Awais et al., 2010). The voltammogram originated by the electrochemical reduction of NiO depends on the method of NiO preparation and deposition (Figure ESI11; Decker et al., 1992; Awais et al., 2010, 2013a).

The most important difference between the electrochemical behaviors of differently deposited NiO films is the degree of reversibility with which the process of electrochemical reduction occurs (Figure ESI11). This aspect depends on the separation of the potential values at which the forward and reverse waves of reduction present a peak of maximum current (Bard and Faulkner, 2001). Since the electrochemical reduction consists in a process of ion intercalation, the observed differences are mostly associated to the differences in the crystal structures and Van der Waals features of the electrode rather than to the morphological characteristics of the oxide surface (Whittingham, 1997, 2000). The most distinctive aspect of the solid-state electrochemical reduction of nanostructured NiO is the dependence of the latter process on illumination with visible light (Awais et al., 2013a): when NiO electrode is irradiated an increase of the current is observed with respect to dark conditions (Figure ESI12). The nature of the electrochemical process of reduction within the potential range 1.25 < *E* < 2.6 V vs. Li^+^/Li is not altered by the illumination since no potential shifts and/or appearance/disappearance of electrochemical waves is observed upon electrode irradiation within that potential range of Figure ESI13. Therefore, the main effect of light irradiation is of photoconductive nature and consists in the increase of the electrical conductivity of illuminated NiO undergoing electrochemical *n*-doping (Equation 5; Passerini and Scrosati, 1994).

An important issue related to the process of reduction of mesoporous NiO films is the eventual involvement of a redox process based on the transparent conductive substrate on which NiO is deposited (Awais et al., 2013b, 2015b). A careful control of the values of applied potential that lead to NiO reduction is then necessary in order to avoid the concomitant reduction of the transparent metallic substrate usually made of ITO or FTO (Awais et al., 2013b, 2015b). In fact, upon stabilization of the voltammogram of bare ITO substrate this supporting metallic conductor undergoes lithium uptake (Cogan et al., 1985; Bressers, 1998; Awais et al., 2013b) within the potential range of NiO electroactivity (Figure ESI13). The reduction of underlying ITO is characterized by the linear dependence of the current peak with the scan rate (Figure ESI13; Awais et al., 2013b) whereas, NiO would display a linear dependence of the current peak of reduction on the square root of the scan rate (Awais et al., 2013a). Such a difference indicates that the reduction of uncovered ITO is kinetically limited by a process of charge transfer localized at ITO surface whereas the reduction of NiO is diffusion controlled. In presence of a non-homogenous layer of NiO the occurrence of ITO reduction (Figure ESI14; Armstrong et al., 1976; Stotter et al., 2005) leads to the complete suppression of the redox activity of NiO with the disappearance of the typical reversible oxidation and reduction waves of NiO and the observation of the sole process of ITO reduction (Figure ESI14; Awais et al., 2013b). The employment of FTO as supporting substrate of nanostructured NiO for the realization of NiO reduction (Equation 5) is certainly more advantageous with respect to ITO. This is because FTO presents electrochemical inertness within the potential range of NiO reduction, and undergoes a process of solid state reduction at more cathodic polarizations with respect to NiO and ITO (Awais et al., 2015a).

## Applications of Nanostructured NiO Electrodes in Photoelectrochemistry

### Light-Driven Electrochemical Production of H_2_ From Water Splitting

Non-fossil fuel H_2_ is formed electrochemically as product of reduction of the H^+^ cation on selected electrodic materials (Holladay et al., 2009) during the electrolytic process of water splitting. The conduction of the same reduction process of H_2_ formation in photoelectrocatalytic conditions (Walter et al., 2010) requires the absorption of light at the photocathode as initial step of activation. Absorption of light generates a separation of charges at the photocathode and the successive reduction of the hydrogen cation will occur provided that the cathode is made of a *p*-type SC. The excess of minority carriers created at the interface that separates the excited *p*-SC from the electrolyte will be responsible of the photoactivated reduction of hydrogen cations. The *p*-SC can possess intrinsic photoelectrocatalytic activity toward H^+^ reduction (Lewerenz et al., 2008; Kargar et al., 2014; Luo et al., 2016; Yang et al., 2016; Zhang et al., 2017). Alternatively, the *p*-SC can achieve such a capability through sensitization with dyes that impart photocatalytic activity to the *p*-SC electrode when the dyes are immobilized on the surface of the p-SC (Tong et al., 2012). In the latter case the *p*-type semiconductor would act as an electron relay toward H^+^, which operates as an heterogeneous catalyst (Muñoz and Lewerenz, 2010; Lewerenz et al., 2011). Reference (Muñoz and Lewerenz, 2010) reports a review of *p*-type semiconducting electrodes for the photoelectrochemical generation of H_2_ when solar radiation is the source of luminous energy (Lewis, 2016). Among nanostructured *p*-SC for photoelectrochemical generation of H_2_ in light-driven water splitting (Gür et al., 2014; Li et al., 2015), NiO in the sensitized state represents the most studied example (Castillo et al., 2015; Dong et al., 2015; Meng et al., 2015; Wood et al., 2016). This is due to the well-established properties as photocathodic material of *p*-DSCs (Dini et al., 2015a; Bonomo and Dini, 2016; Dini, 2016). For the photoelectrocatalytic generation of H_2_ nanostructured NiO cathode can be configured in two main ways: (i) through sensitization with a molecular light absorber which upon optical excitation transfers an electron to a moiety acting as electrocatalytic center for H_2_ formation (Ji et al., 2013; Click et al., 2016; Willkomm et al., 2016); (ii) through sensitization with quantum dots (QDs) made of a semiconducting material with a lower VB edge with respect to nanostructured NiO substrate, with the QDs transferring the optical excitation to a molecular co-catalyst capable of reducing H^+^ for successive H_2_ formation (Meng et al., 2015; Ruberu et al., 2015). These modified NiO electrodes produce H_2_ at rates in the order of 10^−7^ mole per hour and display faradic efficiencies that approximate 100% in conditions of simulated solar irradiation (Ji et al., 2013; Castillo et al., 2015; Dong et al., 2015; Meng et al., 2015; Ruberu et al., 2015; Click et al., 2016; Juodkazyté et al., 2016; Wood et al., 2016). Therefore, the quasi totality of the photogenerated current is exploited for the reduction of H^+^ and side reactions are practically absent. Table 2 reports a list of relevant characteristics of the photoelectrolysis cells which employ nanostructured TMO cathodes (Liao and Carter, 2013) for H_2_ production (Cao et al., 2011; Ji et al., 2013; Rodenas et al., 2013; Liu et al., 2017). The photocathodes are usually decorated with catalysts constituted of supramolecular assemblies the photocatalytic activity of which has been previously verified in homogeneous conditions, i.e., in the non-immobilized state (Rau et al., 2006; Soman et al., 2012; Halpin et al., 2013; Dini et al., 2015b; Pfeffer et al., 2015).

**Table 2:** Semiconducting cathodes based on nanostructured NiO and photoelectrocatalytic agents employed in the potentiostatic generation of H_2_ under simulated solar irradiation.

**Cathode**	**Sensitizer**	**Catalyst**	**Faradic efficiency/%**	**References**
NiO	PMI-6T-TPA	-	97	Tong et al., 2012
NiO	CdSe QDs	Co-based	81	Meng et al., 2015
NiO	CdSe QDs	MoS_2_	100	Dong et al., 2015
NiO	BH4	Mo_X_S_Y_	60	Click et al., 2016
NiO	O22	Co-based	68	Ji et al., 2013
NiO	CdSe	Co(bdf)_2_	100	Ruberu et al., 2015
NiO	C343	Fe-based	50	Antila et al., 2016

**Figure 3:**
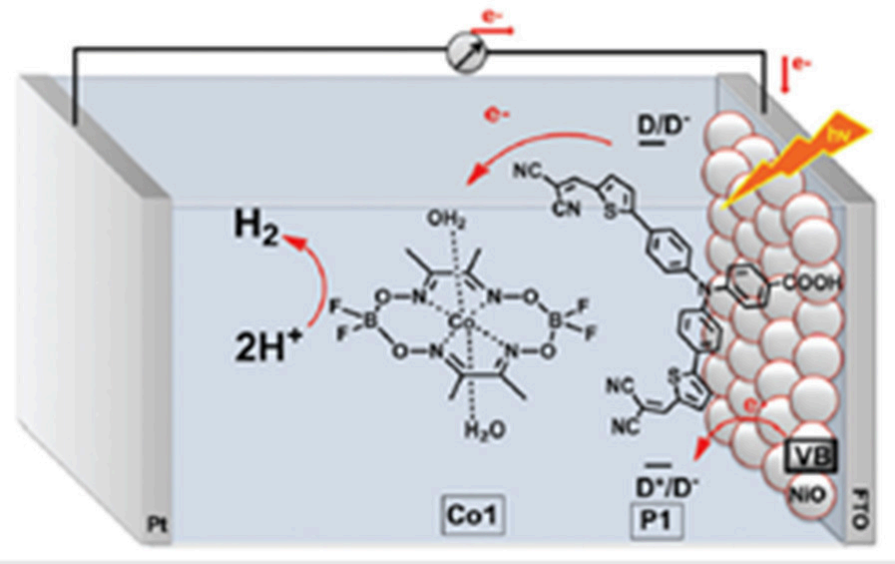
Light-driven production of H_2_ from a PEC of electrolysis, which employs P1-sensitized NiO as photocathode and the co-catalyst Co1 [a Co(II) complex] dissolved in aqueous electrolyte. H_2_ is photogenerated in solution when P1-NiO cathode is illuminated and kept polarized at −0.4 V vs. Ag/AgCl. Reproduced with permission from Zannotti et al. (2015).

A schematic depiction of the working principle operating in the photoelectrolysis cells based on sensitized nanostructured electrodes for the production of solar fuel is shown in Figure 3 when the cathode is made of NiO and the co-catalyst operates in homogeneous conditions (Li et al., 2012).

**Figure 4:**
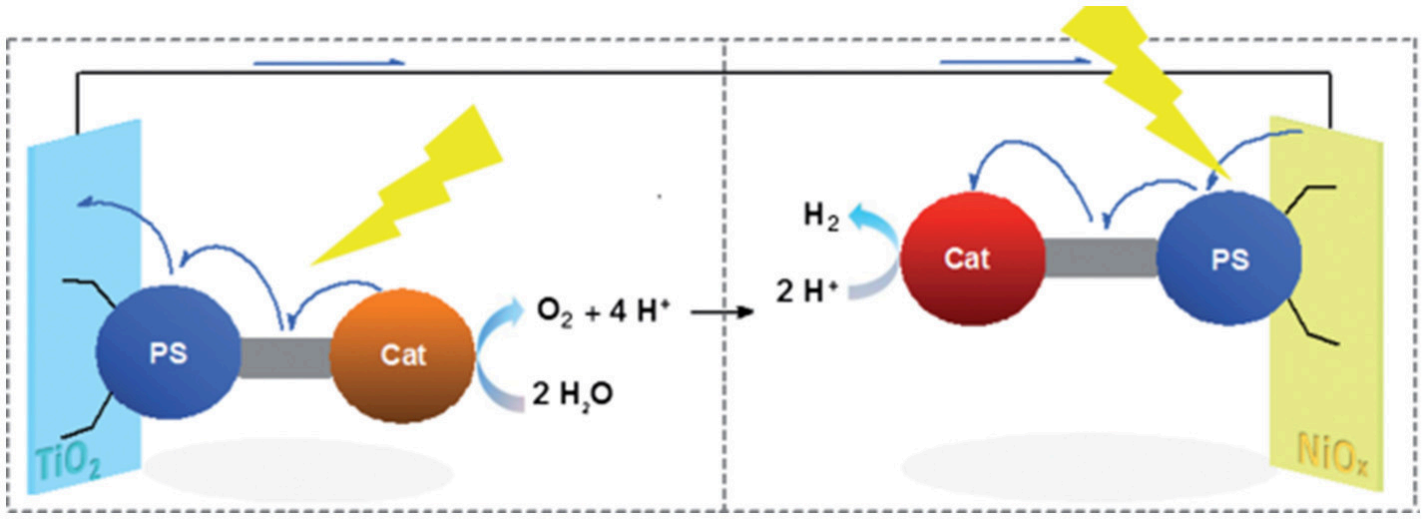
Light-driven water photoelectrolysis with production of H_2_ at the NiO photocathode and O_2_ at the TiO_2_ photoanode. The scheme depicts a PEC in which both oxide electrodes are sensitized with supramolecular assemblies with PS, i.e., the photosensitive moiety of the assembly, and Cat, i.e., the catalytic center of the assembly, which execute a process of *et* upon light excitation of PS. Reproduced with permission from Halpin et al. (2009).

A further evolution in the design of the photocathode for solar driven H_2_ generation is represented by nanostructured TMOs of *p*-type, which are decorated with a supramolecular assembly (Figure 4; Dini et al., 2015b). The latter species combines a photosensitive moiety (PS) electronically conjugated to the catalytic center (Cat) (Halpin et al., 2009, 2010). The whole molecular assembly operates in the surface-immobilized state (heterogeneous mode).

**Figure 5:**
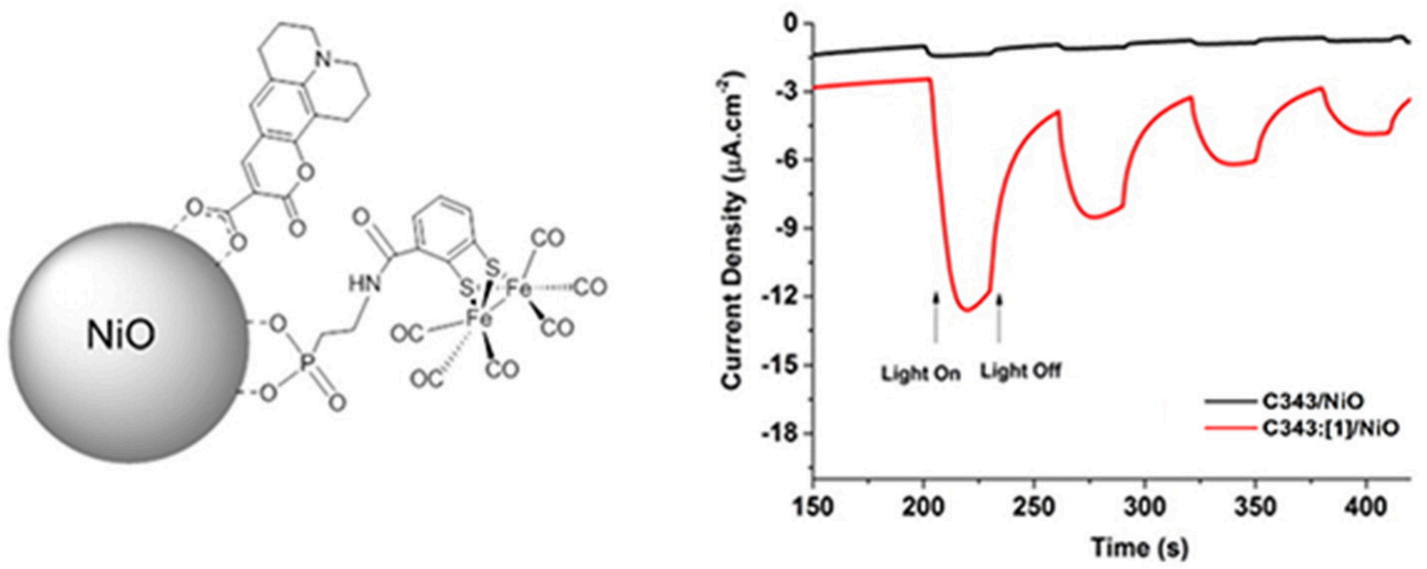
**Left**: depiction of the photoelectroactive material consisting of *p*-type NiO nanoparticles decorated with the dye-sensitizer coumarin 343 (PS) and the biomimetic Fe-Fe catalyst (Cat) directly anchored on NiO through the phosphonate group. **Right**: temporal profiles of the current density associated to H_2_ generation as a function of the light-switching time when Fe-Fe Cat [**1**] is not included (black profile), and when is anchored (red profile). Reproduced with permission from Antila et al. (2016).

The approach described in Figure 4 is particularly attractive since it avoids catalyst replenishment or its *in-situ* regeneration in the liquid electrolyte. Therefore, the PEC design of Figure 4 can allow the realization of photoelectrolysis in the continuous-flow mode (Homayoni et al., 2015) where only the electrolyte is replenished and no expensive and/or time-consuming operations of separation/purification are involved. The practical realization of the PEC operating has been reported by Antila et al. (Figure 5) when the molecular assemblies comprised a PS unit of coumarin 343 and a Cat unit of Fe-Fe biomimetic catalyst (Antila et al., 2016).

In this configuration the PS and Cat units of the photoelectrocatalyst interact electronically through space and not conjugated moiety, e.g., a bridging ligand that connects the two units, is involved. The resulting Faradaic efficiency of this modified NiO electrode was about 50% (Antila et al., 2016).

### Light-Driven Electrochemical Reduction of CO_2_

**Figure 6:**
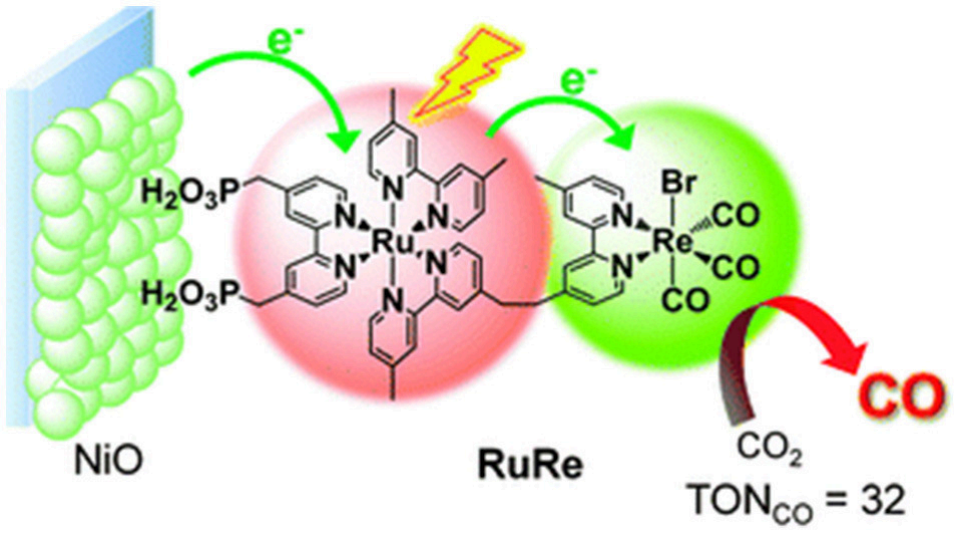
Definition of a photocathode based on nanostructured NiO for the selective photoelectrochemical reduction of CO_2_ to CO. The photocathode is sensitized by a di-nuclear complex of Ru and Re having the dual function of absorbing visible light and transferring electrons to CO_2_. Reproduced with permission from Takeda and Ishitani (2010).

**Figure 7:**
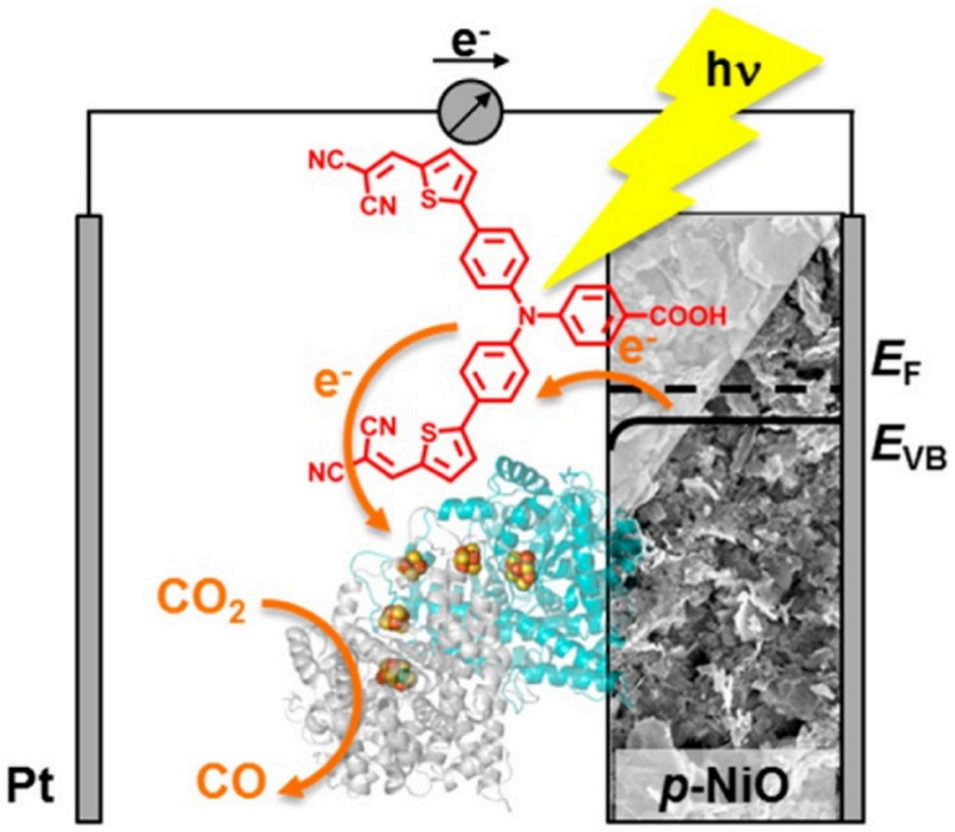
Scheme of the photoelectrocatalytic action exerted by a dye-sensitized NiO cathode decorated with the enzyme *carbon monoxide dehidrogenase I* toward the reduction of CO_2_ to CO. The light absorbing unit is P1 (in red). Reproduced with permission from Bachmeier et al. (2014).

For the mitigation of the environmental effects related to the anthropogenic formation of CO_2_ several approaches have been proposed (Aresta et al., 2014). These consist generally in the transformation of CO_2_, i.e., an abundant and polluting component of the terrestrial atmosphere, into useful products through procedures that should be at very low environmental impact during operation. The photoelectrochemical reduction of CO_2_ on photoactive sensitized cathodes (Barton et al., 2008; Xie et al., 2015) has been demonstrated and resulted particularly attractive for a series of important reasons (Sakakura et al., 2007) like the transformation of CO_2_ into fuels (CH_4_, CH_3_OH). At a large scale the conduction of the latter process through the photoelectrochemical approach would ideally diminish the request of fossil fuels extracted from fields. It has been recognized that the photoelectrochemical reduction of CO_2_ proceeds efficaciously through two paths (Herron et al., 2015) (a) by means of the primary photoelectrochemical production of H_2_ (a strong reductant) and its successive reaction with CO_2_ to give selectively hydrogenated products with high energy density or synthetic usefulness; (b) by means of the direct photoelectrochemical reduction of CO_2_ (Aresta et al., 2014). In a recent review (Bonomo and Dini, 2016) simple calculations and basic considerations have shown that NIR and visible light do not deliver an energy sufficiently high to initiate directly any kind of reaction of CO_2_ which involves the initial rupture of a C = O bond. This is because the minimum energy threshold for the removal of one electron from CO_2_, i.e., the step that would start the breaking of one of the two covalent CO bonds and allow any further chemical transformation of CO_2_, is about 4 eV. Such a value of energy corresponds to a radiation wavelength of about 300 nm, i.e., a value which falls typically in the UV range. On the other hand, visible light can result useful in case of the process of mono-addition of an electron in the LUMO of CO_2_ (which would thus become CO${}_{2}^{-}$ ; Bonin et al., 2014b) provided that an opportune electron donor is present at a tunneling distance from neutral CO_2_. A *p*-SC [either in the pristine or dye/co-catalyst modified state (Vesborg and Seger, 2016)] can achieve the single electron addition to CO_2_ if *p*-SC is illuminated with visible light. For the improvement of the kinetics of reduction at a *p*-SC cathode one has to increase the energy of the electrons till their promotion to the CB of the *p*-SC (Harris and Wilson, 1978). In doing so the electrons will be transferred to the oxidized form of the redox couple at a diminished activation energy. The energy of electrons at a *p*-SC electrode can be modulated by means of the application of a cathodic external bias, a cathodic electrical current and upon direct/indirect/mediated absorption of the luminous radiation (Begum and Pickup, 2007; Angamuthu et al., 2010; Kas et al., 2014). Under opportune conditions of illumination the utilization of a sensitized *p*-SC electrode for the photoelectrochemical reduction of CO_2_ (Windle et al., 2015) avoids the use of a sacrificial agent (Bonin et al., 2014a) since the photoelectrochemical process is conducted in the heterogeneous modality with the electrons that are replenished by the photocurrent passing in the cell. The photoelectrochemical reduction of CO_2_ via a surface-immobilized molecular assembly (Figures 6, 7) that accomplishes the dual function of light absorption (through PS) and electron(s) transfer (*et*) to the electrocatalytic moiety (Cat) interacting directly with CO_2_, implies the realization of the following sequence of elementary steps:(Kumar et al., 2012).

- Excited state formation of the PS moiety with consequent charge separation;
- Intramolecular *et* from the excited PS moiety to Cat with the latter coordinating CO_2_;
- Step of *et* from Cat (acting as electron relay) to CO_2_;
- Uptake of one or more electron from *p*-SC (acting as regenerator of the assembly thanks to the passage of an electrical current of electrolysis), for the neutralization/regeneration of the PS moiety and/or the Cat unit that resulted temporarily oxidized for the occurrence of the previous steps (b) and (c).

The most interesting examples of nanostructured TMOs of *p*-type for the efficacious photoelectrochemical reduction of CO_2_ are the combination of Cu_2_O/CuO in the shape of nanorods (Rajeshwar et al., 2013), and native SnO_*x*_ onto Sn substrate (Magesh et al., 2014). Both systems did not make use of any auxiliary supramolecular assembly since they presented intrinsic self-absorption in the NIR-visible range combined to optimal charge transport properties (Hinogami et al., 1998). The Cu-based mixed oxide could convert CO_2_ into methanol CH_3_OH with 95% of faradic efficiency when it was kept polarized at −0.20 V vs. SHE under one sun of illumination. The use of the photoelectrode Sn/SnO_*x*_ led to the formation of formic acid HCO_2_H as main product of photoelectrochemical reduction of CO_2_ in potentiostatic conditions with a resulting faradic efficiency of 27.5 % at + 0.70 V vs. SHE. An example of sensitized photocathode for CO_2_ electrochemical reduction is the one obtained upon sensitization of nanostructured NiO with a di-nuclear complex of Ru and Re known for having photocatalytic properties (Braumüller et al., 2016; Nakada et al., 2016). Such a system (Figure 6; Nakada et al., 2016) resulted photoelectrochemically active toward the selective reduction of CO_2_ to carbon monoxide CO at the potential of polarization −1.2 V vs. Ag/AgCl.

In the example of Figure 6, it remains still an open question the definition of the actual role of the CO ligand coordinated by Re(I) in the process of CO formation from CO_2_ (Takeda and Ishitani, 2010). Moreover, the capability of NiO to transfer electrons neatly toward the Re(I) center through a Ru(II) bipyridyl complex is not so obvious given the scarce matching of the energy levels between *p*-type NiO and N719 or Black Dye (Nattestad et al., 2008; Novelli et al., 2015; Sheehan et al., 2015), i.e., two complexes that are structurally very similar to the light absorbing unit of the RuRe complex in Figure 6. The structure of the RuRe assembly could be at the basis of the relatively low turnover number (TON) of 32 found by that authors. In fact, the equivalent amount of sensitizer typically chemisorbed on the surface of nanostructured NiO (Powar et al., 2012) would give a much higher efficiency of CO_2_ photoconversion when employed in the non-immobilized state (Takeda and Ishitani, 2010). Another photoelectrocatalytic system based on dye-sensitized nanostructured NiO for the photoelectrochemical reduction of CO_2_ is the one proposed as a proof of concept by Bachmeier et al. (2014) (Figure 7).

The photoelectrocatalytic cathode of Figure 7 is constituted by the P1-sensitized skeleton of nanostructured NiO. In this configuration the NiO is also modified by the enzyme *carbon monoxide dehidrogenase I* that adsorbs spontaneously on NiO surface and acts as catalytic unit in the transformation of CO_2_ into CO. Alike the photoelectrocatalytic system depicted in Figure 6, the enzyme-modified NiO electrode for the photoreduction of CO_2_ (Figure 7; Bachmeier et al., 2014) operates with the PS and Cat units accomplishing the *et* process through space and not through bonds.

## Concluding Remarks

This review has given an overview on the electrochemical and photoelectrochemical behavior of semiconducting electrodes made of nanostructured transition metal oxides (TMOs) like NiO in the configuration of thin films. The interest in NiO resides primarily in the chemical-physical stability which is imparted by bonds having mixed covalent and ionic character. Such a combination of characters generates an electronic structure characterized by the presence of energy bands and partially delocalized states at the valence level with impartment of semiconducting properties. TMO based semiconductors like NiO are photoactive since such electrode materials can transfer electrons in the desired direction provided that a radiation of opportune energy is absorbed by the TMOs for the primary realization of the hole-electron separation. Unlike the semiconductors based on Si, NiO undergoes reversible electrochemical redox processes in the solid state (either in the dark or illuminated states), and as such represent electroactive species. The occurrence of a NiO-based redox process leads to the doping of the oxide and it is accompanied by charge storage. The latter process is also of great utility for the development of batteries and primary sources of electrical energy based on NiO electrodes. Nanostructuring of NiO is an efficacious tool for modulating of the electrochemical properties, the optical absorption and the characteristics of charge transport. Therefore, the preparation of nanosized NiO gives further opportunities for employing NiO as photoelectroactive materials for the finalities of solar energy conversion (with the development of dye-sensitized solar cells, DSCs), solar fuels generation during water photoelectrolysis, and the photoelectrochemical reduction of CO_2_. In DSCs TMOs have demonstrated to be already a quite mature choice having offered overall conversion efficiencies of 14, 2.55, and 1.70%, respectively, in the DSCs of *n*-type, *p*-type, and tandem type. Many factors other than the nature of TMOs affect the performances of DSCs, but room for the improvement of the synthetic and deposition procedures of TMOs is still ample for the amelioration of the performances of these devices. For the development of photoelectrochemical cells for H_2_ photogeneration, water photosplitting and CO_2_ reduction, the approach is the same of the DSCs (Grätzel cells) with the adoption of dye-sensitized nanostructured NiO of *p*-type as photoelectroactive component. Different to DSC, it has been early recognized that the sole light-absorbing unit in the immobilized state is not sufficient to accomplish the photoelectrochemical reduction of H^+^ for the successive formation of H_2_ during water splitting (or the reduction of CO_2_) due to the complexity and the number of chemical reactions that follow the starting electrochemical step of electron transfer. For this reason, the successive development of catalytic units was necessary for the full realization of the wanted photoreduction process at modified NiO. At the present stage the progress on the photoelectrolysis cells for the realization of photoelectrocatalytic reduction processes depends mainly on the nature of the dye-sensitizer and the catalytic units combined in the multifunctional supramolecular assembly PS-Cat (either bridged or separated) rather than on semiconducting NiO cathode., The special attention given by the present review to the analysis of the electrochemical and photoelectrochemical properties of *p*-type NiO is due to the variety of the (photo)electrochemical processes occurring in NiO electrodes, and the complexity of the kinetics of NiO redox processes. Both characteristics certainly render this system a paradigmatic example for the class of semiconducting TMO electrodes.

## Author Contributions

All authors contributed equally in the compilation of the text, the bibliographic analysis and the preparation of figures and tables. The content of the present contribution has been approved by all authors.

### Conflict of Interest Statement

The authors declare that the research was conducted in the absence of any commercial or financial relationships that could be construed as a potential conflict of interest. The reviewers TS, IP and handling Editor declared their shared affiliation.
