# Supplementary material for: Electrochemical and Photoelectrochemical Properties of Nickel Oxide (NiO) With Nanostructured Morphology for Photoconversion Applications
Source: Front Chem. 2018 Dec 12;6:601. doi: 10.3389/fchem.2018.00601 (PMC6299045; doi:10.3389/fchem.2018.00601)
Supplement: Supplementary file 1 [file Data_Sheet_1.docx]

**SUPPLEMENTAL MATERIAL**

**Review title: Electrochemical and photoelectrochemical properties of nickel oxide (NiO) with nanostructured morphology for photoconversion applications**

**Authors:** Matteo Bonomo, Danilo Dini, Franco Decker

**Figure ESI1.** Cyclic voltammetry of NiO prepared via plasma-assisted rapid discharge sintering of sprayed NiO nanoparticles with an average diameter of 50 nm. Substrate: indium-doped tin oxid (ITO); electrolyte composition: 0.7 M LiClO_4_ in anhydrous propylene carbonate (PC); counter electrode: Li; reference electrode: Li^+^/Li; scan rate: 5 mV s^-1^. NiO thickness: 0.3 μm. Adapted from ref. ESI1.

**
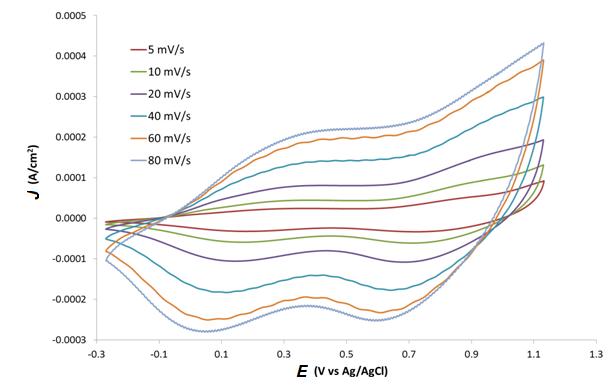

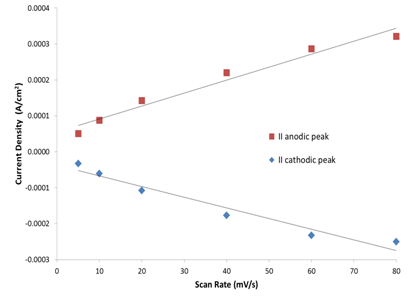
**

**Figure ESI2.** Left: Voltammograms of bare NiO (*l* = 2.5 μm) at different scan rates (electrolyte: 0.2M LiClO_4_ in 3-methoxy-proprionitrile). Right: scan rate dependence of the amplitude of the anodic and cathodic current peaks associated to the redox process of Eq.2. Reprinted with permission from ref. ESI2.

**Figure ESI3.** Voltammograms of two NiO electrodes differing for the thickness of the film. Scan rate : 20 mV s^−1^. Electrolyte: 0.2 M LiClO_4_ in 3-methoxy-proprionitrile. The blue arrows indicate the verse of potential scan. Reproduced from ref. ESI3 (Open Access Source).

**
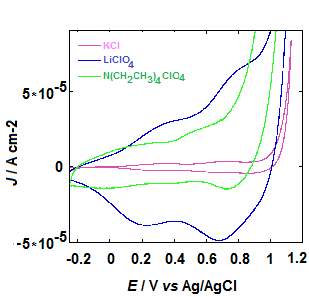
**

**Figure ESI4.**  Left: effect of the nature of the cation of the supporting electrolyte on the shape of the voltammograms associated to the oxidation of nanostructured NiO (scan rate 10 mV s^-1^). Anion is triflate. Electrolyte concentration is 0.2 M in 3-methoxy-propionitrile. Right: effect of the nature of the anion and cation of the supporting electrolyte on the same electrochemical process of the left frame (scan rate 2 mV s^-1^). Right frame has been reproduced from ref. ESI4 (Open Access Source). Left frame has been reproduced with permission from ref. ESI4. Copyright 2001. American Chemical Society.

**Figure ESI5.** Effect of white light illumination (*I_in_*: 25 W cm^-2^) on the oxidation of nanostructured NiO sensitized with erythrosine b. Both cyclic voltammetries were conducted at the scan rate of 25 mV s^-1^. Voltammograms were recorded after stabilization (Figure ESI7, left plot, *vide infra*). Reproduced from ref. ESI5 (Open Access Source).

**Figure ESI6.** Effect of the sensitization of nanostructured NiO (*l* = 2 μm) with a squaraine on the process of electrochemical oxidation of NiO analyzed with the technique of cyclic voltammetry (electrolyte: 0.2 M LiClO_4_ in 3-methoxy-propionitrile; scan rate: 20 mV s^-1^). Reproduced with permission from ref. ESI2.

**Figure ESI7.** Left: evolution of the voltammogram of nanostructured NiO sensitized with erythrosine b. The potential was scanned with the cell in dark conditions (scan rate: 5 mV s^-1^). Right: effect of the scan rate on the voltammogram of erythrosine-sensitized NiO. Voltammograms were taken at different scan rates after stabilization of the voltammogram recorded at 5 mV s^-1^ (left plot). Reproduced with permission from ref. ESI1.

**Figure ESI8.** Dependence of the voltammogram of NiO sensitized with erythrosine b on the scan rate when the electrode is illuminated with white light (*I_in_*: 25 W cm^-2^). Reproduced with permission from ref. ESI1.

**
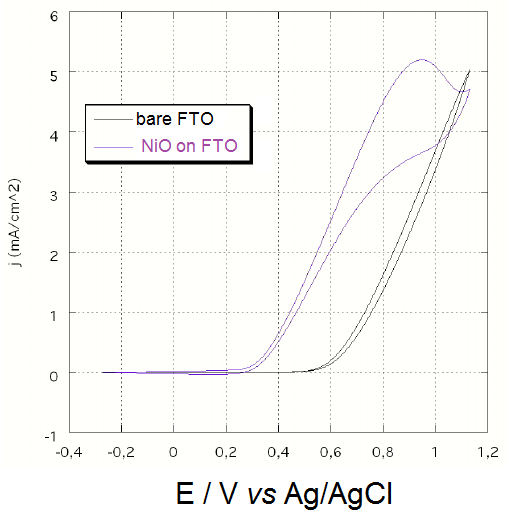

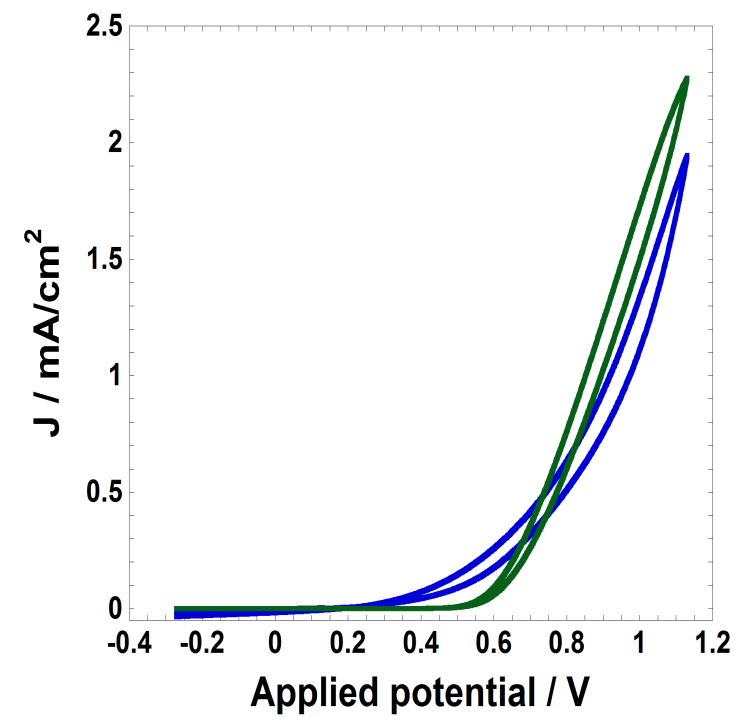
**

**Figure ESI9.** Electrocatalytic effect of nanostructured NiO electrode *vs* FTO substrate towards iodide oxidation (3I^-^ → I_3_^-^ + 2 *e*^-^). Left: comparison of the voltammograms associated to I^-^ oxidation, which have been recorded with rapid discharge sintered NiO electrode (*l* : 2.5 μm) and bare FTO at the scan rate of 20 mV s^-1^. Right: (blue trace) voltammogram recorded with a bare NiO electrode (*l* : 4 μm) prepared via screen printing; (green trace) voltammogram recorded with uncovered FTO as electrode. Scan rate: 10 mV s^-1^. Potential is referred to the redox couple Ag/AgCl. Electrolyte (composition: 0.2 M LiI, 0.02 M I_2_ in acetonitrile) was the same in both experiments with the two different NiO samples. Left frame is reproduced with permission from ref. ESI2. Right frame has been reproduced with permission from ref. ESI4 Copyright 2016. American Chemical Society.


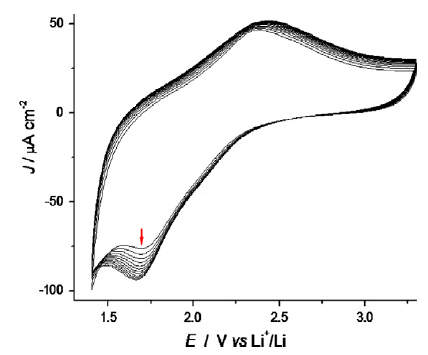


**Figure ESI10.** Variation of the voltammograms of microblast deposited NiO (*l* = 1.2 μm) associated to the solid state reduction of Eq.5 upon repetitive cyclying of potential (scan rate: 40 mV s^-1^). Electrolyte composition: 0.5 M LiClO_4_ in propylene carbonate. Red downward arrow indicates the increase of the current peak upon repeated cycling. Reproduced with permission from ref. ESI5.


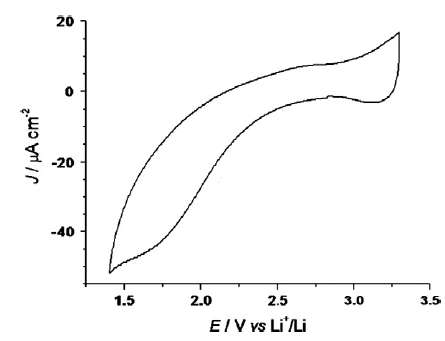


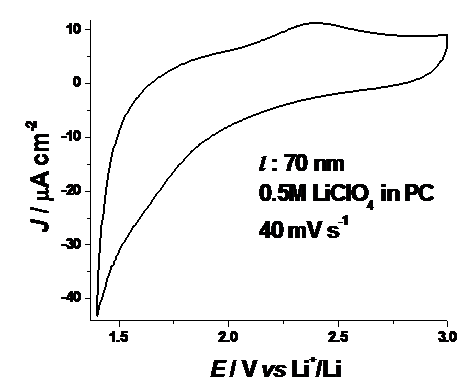


**Figure ESI11.** Effect of the deposition method on the reduction process of NiO (Eq. 5) in anhydrous electrolyte. Top: voltammogram of a conventionally sintered NiO sample (*l* = 1.2 μm) at the scan rate of 40 mV s^-1^. Bottom: voltammogram of a NiO sample deposited via magnetron sputtering. Electrolyte composition: 0.5 M LiClO_4_ in propylene carbonate (PC) for both experiments carried out with the two differently deposited NiO samples. Reproduced with permission from refs. ESI6 and ESI7.


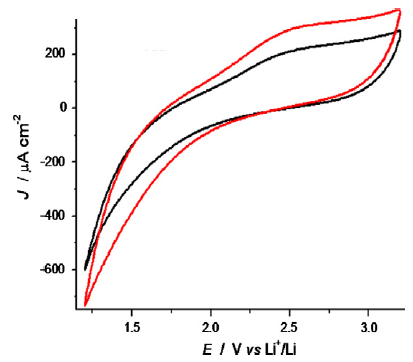


**Figure ESI12.** Effect of white light illumination on the cyclic voltammogram of microblast NiO (*l* = 1.2 μm) in correspondence of the reduction process described in Eq.5 (see MainText). The black and red curves refer to the voltammograms recorded respectively in dark conditions and under illumination with an halogen lamp (*P*_in_ = 500 W). Reproduced with permission from ref. ESI8.

**Figure ESI13.** Left: stabilized cyclic voltammogram of bare ITO after 1000 cycles within the potential range 1.2-2.8 V *vs* Li^+^/Li (scan rate: 7 mV s^-1^). Electrolyte: 0.7 M LiClO_4_ in anhydrous propylene carbonate. Red arrows indicate the verse of potential scan. Right: voltammograms of bare ITO conducted at different scan rates after electrochemical stabilization (left plot). Reproduced with permission from ref. ESI9.

**Figure ESI14.** Voltammogram of a NiO-covered ITO substrate recorded after electrochemical stabilization in the experimental conditions of Figure ESI14. Scan rate: 15 mV s^-1^. Adapted from ref. ESI10 (Open Access Source).

**References**

**ESI1.** D. Dini, Y. Halpin, J.G. Vos, E.A. Gibson, *Coord. Chem. Rev.*, 2015, **304-305**, 179

**ESI2.** S. Sheehan, G. Naponiello, F. Odobel, D.P. Dowling, A. Di Carlo and D. Dini, *J. Solid State Electrochem.*, 2015, **19**, 975

**ESI3.** V. Novelli, M. Awais, D. P. Dowling and D. Dini, *Am. J. Anal. Chem.*, 2015, **6**, 176

**ESI4.** G. Boschloo and A. Hagfeldt, *J. Phys. Chem. B*, 2001, **105**, 3039

**ESI5.** M. Awais, D. D. Dowling, F. Decker and D. Dini, *Springerplus*, 2015, **4**, 564

**ESI6.** M. Bonomo, D. Dini and A. G. Marrani, *Langmuir*, 2016, **32**, 11540

**ESI7.** M. Awais, D. Dini, J. M. Don MacElroy, Y. Halpin, J. G. Vos and D. P. Dowling, *J. Electroanal. Chem.*, 2013, **689**, 185

**ESI8.** M. Awais, M. Rahman, J. M. Don MacElroy, N. Coburn, D. Dini, J. G. Vos and D. P. Dowling, *Surf. Coatings Technol.*, 2010, **204**, 2729

**ESI9.** M. Awais, D. D. Dowling, M. Rahman, J. G. Vos, F. Decker and D. Dini, *J. Appl. Electrochem.*, 2013, **43**, 191

**ESI10.** M. Awais, D.P. Dowling, F. Decker and D. Dini, *Adv.Cond. Matt. Phys.*2015, **2015**, 186375
